# YPTB3816 of *Yersinia pseudotuberculosis* strain IP32953 is a virulence-related metallo-oligopeptidase

**DOI:** 10.1186/s12866-016-0900-7

**Published:** 2016-11-25

**Authors:** Ali Atas, Alan M. Seddon, Donna C. Ford, Ian A. Cooper, Brendan W. Wren, Petra C. F. Oyston, Andrey V. Karlyshev

**Affiliations:** 1 School of Life Sciences, Pharmacy and Chemistry; Faculty of Science, Engineering and Computing, Kingston University, Penrhyn Road, Kingston upon Thames, KT1 2EE UK; 2 Department of Pathogen Molecular Biology, London School of Hygiene and Tropical Medicine, Keppel Street, London, WC1E 7HT UK; 3 Biomedical Sciences, DSTL Porton Down, Salisbury, Wiltshire SP4 0JQ UK

**Keywords:** *Yersinia pseudotuberculosis*, Proteases, Proteolysis, Oligopeptidases, Metallopeptidases, Virulence

## Abstract

**Background:**

Although bacterial peptidases are known to be produced by various microorganisms, including pathogenic bacteria, their role in bacterial physiology is not fully understood. In particular, oligopeptidases are thought to be mainly involved in degradation of short peptides e.g. leader peptides released during classical protein secretion pathways. The aim of this study was to investigate effects of inactivation of an oligopeptidase encoding gene *opdA* gene of *Yersinia pseudotuberculosis* on bacterial properties in vivo and in vitro, and to test dependence of the enzymatic activity of the respective purified enzyme on the presence of different divalent cations.

**Results:**

In this study we found that oligopeptidase OpdA of *Yersinia pseudotuberculosis* is required for bacterial virulence, whilst knocking out the respective gene did not have any effect on bacterial viability or growth rate in vitro. In addition, we studied enzymatic properties of this enzyme after expression and purification from *E. coli*. Using an enzyme depleted of contaminant divalent cations and different types of fluorescently labelled substrates, we found strong dependence of its activity on the presence of particular cations. Unexpectedly, Zn2+ showed stimulatory activity only at low concentrations, but inhibited the enzyme at higher concentrations. In contrast, Co2+, Ca2+ and Mn2+ stimulated activity at all concentrations tested, whilst Mg2+ revealed no effect on the enzyme activity at all concentrations used.

**Conclusions:**

The results of this study provide valuable contribution to the investigation of bacterial peptidases in general, and that of metallo-oligopeptidases in particular. This is the first study demonstrating that *opdA* in *Yersinia pseudotuberculsosis* is required for pathogenicity. The data reported are important for better understanding of the role of OpdA-like enzymes in pathogenesis in bacterial infections. Characterisation of this protein may serve as a basis for the development of novel antibacterials based on specific inhibition of this peptidase activity.

**Electronic supplementary material:**

The online version of this article (doi:10.1186/s12866-016-0900-7) contains supplementary material, which is available to authorized users.

## Background

Proteases are ubiquitous enzymes found in both eukaryotes and prokaryotes and play a pivotal role in many biological functions [1, 2]. Oligopeptidases, such as OpdA, OpdB and Dcp, specifically cleave short peptides and are inactive against full-length large proteins such as casein [1]. OpdA (or PrlC [3]) is a cytoplasmic zinc-dependent oligopeptidase belonging to the M3A subfamily of proteases containing a highly conserved alpha-helix forming zinc binding motif HEXXH located in a channel restricting access of proteins [4]. The enzymatic activity requires binding of the histidine residues with a zinc cation, and the glutamic acid residue carries out the catalytic role [5]. OpdA was first identified in *Salmonella enterica* serovar Typhimurium as an enzyme capable of hydrolysing *N-*acetyl-L-alanyl-L-alanyl-L-alanyl-L-alanine (AcAla_4_) [6], and subsequently was also identified in *Escherichia coli* [1, 7, 8]. A tyrosine residue Y607 was identified as a key residue in substrate recognition [7].

OpdA and some eukaryotic oligopeptidases, such as thimet oligopeptidase (TOP) and neurolysin, share the same zinc binding motif HEFGH and some amino acid sequence similarity and can be differentiated by substrate specificity [8]. In contrast to mammalian oligopeptidases, the biological role of OpdA is not well understood and has only been investigated for *E. coli* and *S.* Typhimurium. Diverse functions have been suggested for the enzyme, such as signal peptide break down [1, 9], downstream hydrolysis of peptides for amino acid recycling [1], and the roles in the development of phage P22 in *Salmonella* Typhimurium [10] and in a heat shock response [11].

Apart from *S.* Typhimurium and *E. coli* there have been no reports on the characterization of OpdA in other bacterial species. The aim of this study was to characterize an OpdA homologue in a further member of the Enterobacteriaceae, *Yersinia pseudotuberculosis*. Hosts infected with this pathogen show symptoms of mesenteric lymphadenitis, gastroenteritis and septicemia in a disease termed yersiniosis [12]. We show here that inactivation of a putative OpdA encoding gene in *Y. pseudotuberculosis* IP32953 (YPTB3816, also annotated as *prlC*, GenBank accession number CAH23054.1) resulted in attenuation in a murine model of infection, suggesting a role for OpdA in the pathogenesis of *Y. pseudotuberculosis*. The derived amino acid sequence of this protein revealed 81% identity (100% coverage) with that of *E. coli* OpdA (GenPept accession number NP_417955) suggesting some similarity in their functions. The recombinant OpdA of *Y. pseudotuberculosis* was purified, and its catalytic activity was characterized.

## Methods

### Bacterial strains and growth conditions

*E. coli* XL1 Blue and *Y. pseudotuberculosis* IP32953 strains were grown on Luria Bertani plates or in LB broth at 37 or 28 °C respectively. When required, the media were supplemented with kanamycin (50 μg/ml) or chloramphenicol (10 μg/ml).

### Construction of the *Y. pseudotuberculosis ΔopdA* mutant

Mutagenesis of *Y. pseudotuberculosis* was performed as described previously [13, 14]. Briefly, a PCR product, containing the kanamycin resistance gene from plasmid pUC4K and flanking regions corresponding to the 5′ and 3′ proximal parts of *opdA*, was generated using the following primers: Yptb3816_kan_for (CCGTTCTCCCTGCCACCGTTTTCTGCTATTCGGCCTGAAGATATCGTGCCCACAGGAAACAGCTATGACC) and Yptb3816_kan_rev (GCAACATGGCATCTAACTGCGGTTCACGGCCACGGAAGCGTTTGAACAGTCAAGTCAGCGTAATGCTCTGC). The PCR product was transformed into *Y. pseudotuberculosis* IP32953/pAJD434 by electroporation. Transformants were verified by PCR using screening primers Yptb3816_for (ATGACAAACCCGCTGTTGACT) and Yptb3816_rev (TTAGCCCTTAATACCGTAATGAC) (Additional file 1: Figure S1). The mutant was cured of the helper plasmid, and the presence of the virulence plasmid pYV was confirmed using primers yscU-for (TCTGTACTGTTGGCTTTGTGC) and yscU-rev (TTGCGCACAGTCTGAACTTGG). The procedure resulted in a deletion of 98% of the *opdA* gene.

### Effect of *opdA* mutation on bacterial fitness in vivo

Six to eight week old female BALB/c mice were obtained from a commercial supplier (Charles River, United Kingdom). On arrival, mice were housed in groups of 5 in polypropylene solid bottom cages with a wire mesh lid, integral diet hopper and water bottle holder (M3, NKP cages, Coalville, UK) within a UK Advisory Committee on Dangerous Pathogens (ACDP) level 3 isolator and allowed to acclimatize before experimental use. Mice were provided with ad libitum irradiated water and ad libitum irradiated diet (5002 Certified Rodent Diet, LabDiet, St Louis, Missouri, USA). Mice were provided with corn cob bedding (1014 Corn Cob, IPS Product Supplies Ltd, London, UK) with enrichment provided as a dome home (LBS Biotech, Crawley UK), aspen wood wool (LBS Biotech, Crawley, UK) and hemp fiber mat (Happi-Mat, Marshall Bio-Resources, Hull, UK). Lighting cycle was 12 h light, 12 h dark with environmental temperatures and humidity maintained within the specified range for rodents under ASPA. Mice were checked a minimum of twice daily, with clinical signs observed, scored and recorded and used to apply the humane endpoint specified in the project license for mice challenged with *Yersinia* spp. Mice were observed at least twice daily for end-point criteria, including loss of appetite, hunched posture, gait and righting difficulty, prostration, ruffled fur and gummy eyes. The animals that reached end-point criteria and animals that survived through the end of the experiment were humanly euthanized by cervical dislocation.

In vivo competitive index (CI) studies were performed as described in [13]. Briefly, mutant and wild type strains were grown separately to exponential phase, cells deposited by centrifugation and the pellet washed once with sterile PBS. The bacteria were re-suspended in PBS and the OD_600_ adjusted to 0.55–0.60. Wild type and mutant bacterial suspensions were then mixed in a 1:1 ratio and serially diluted with sterile PBS to produce bacterial suspension with approximately 1 × 10^3^ cfu/ml. Groups of six mice were then dosed with 0.1 ml of bacterial suspension by the intravenous (i.v.) route. Retrospective viable counts were determined by plating out dilutions (in triplicate) on LB agar and LB agar supplemented with kanamycin to determine the input ratio. Mice were killed by cervical dislocation on day 5, with spleens collected after confirmation of death. Spleens were passed through 70 μm sieves (Becton Dickinson) to produce a cell suspension in 3 ml of PBS. Cell suspensions were serially diluted in sterile PBS and plated onto LB agar and LB agar supplemented with kanamycin to determine the output ratio. The CI is defined as the output ratio (mutant/wild type) divided by the input ratio (mutant/wild type) [15].

### Testing effect of *opdA* mutation on bacterial growth rate in vitro

For growth curves, bacteria were suspended to an OD_590_ of 0.05 in 50 ml LB broth and incubated with shaking (200 rpm) overnight at 28 °C. Both wild-type and mutant strains grew to a similar density during overnight incubation. The bacteria were pelleted by centrifugation and washed once with LB broth before being re-suspended to an OD_590_ of 0.05 in L-broth. Growth curves were performed in a 96 well microtitre plate format. Outer wells were filled with 200 μl distilled water to reduce evaporation and test wells with 200 μl of each test culture. Each strain was tested three times each with six technical replicates. A sterile gas permeable membrane (Breathe-Easy, Diversified Biotech) was used to seal the 96-well plates. Growth curves were generated using a microplate reader (Multiskan FC, Thermo Scientific) housed in a class II biological safety cabinet. The plate was incubated at 28 °C with shaking at 5 Hz, amplitude 15 mm and the OD_595_ recorded every 15 min for 24 h.

### Expression and purification of OpdA

Genomic DNA of *Y. pseudotuberculosis* IP32953 was used as a template to amplify the *opdA* gene using PCR. The primers used to amplify the gene were: GATTCTAGAAGAAGGAGATATACCATGCATCATCATCATCATCACACAAACCCGCTGTTGACTCCGTTCTCCCTG (forward) and TTAGCCCTTAATACCGTAATGACGCAAC (reverse). Nucleotides underlined indicate the introduced restriction site for *Xba*I required for sub-cloning. The amplified 2.1 kb fragment was cloned into the pGEM-T Easy vector (Promega) using the manufacturer’s instruction. After sequence verification, the *Xba*I/*Sph*I fragment was subcloned into the expression vector pBAD33 [16] to produce plasmid pBAD33opdA-His. The derived recombinant plasmid encoded a full copy of OpdA protein with an N-terminal 6xHis tag.

*E. coli* XL1 Blue (Stratagene) strain carrying pBAD33opdA-His was grown at 37 °C in LB broth (Sigma) supplemented with 10 μg/ml chloramphenicol (Sigma). Expression of OpdA fusion protein (referred to as OpdA in this article) was induced at an optical density at 600 nm of 0.6–0.7 by addition of 0.1% *w/v* L-arabinose (Acros Organics) followed by incubation for 1 h at 37 °C. The protein was purified according uning Ni^2+^-NTA Fast Start Kit (Qiagen) according to manufacturer’s protocol. Briefly, the cells were harvested by centrifugation and incubated for 30 min on ice with lysis buffer (Qiagen) containing 50 mM monosodium phosphate, pH 8.0, 300 mM NaCl, 10 mM imidazole and 3 μg lysozyme. After removal of the cellular debris by centrifugation at 4000 rpm for 30 min at 4 °C, the cleared lysate was loaded onto a Ni^2+^-NTA Fast Start Kit column (Qiagen), and the enzyme was purified under native conditions following the manufacturer’s protocol. Proteins were analyzed using sodium dodecyl sulfate polyacrylamide gel electrophoresis (SDS-PAGE). The eluate (1 ml) was dialyzed at room temperature against phosphate buffered saline (PBS) (1000 ml) using 3.5 kDa dialysis tubing (Sigma) to remove imidazole that may have interfered with the enzyme assays. Protein concentration was determined using bicinchoninic acid assay (BCA) kit (Fisher Scientific). The dialyzed protein was stored at 4 °C.

### Enzymatic assays

In this study we used internally quenched fluorogenic substrates, containing Abz (fluorophore) and EDDnp (quencher) at the N-terminal and C-terminal ends respectively. Fluorescence is induced upon digestion of the peptide, followed by separation of the fluorophore from the quencher. Fluorescence of the products of hydrolysis of Abz-NKPRRPQ-EDDnp and Abz-AAL-EDDnp substrates (LifeTein) by OpdA was measured at 37 °C in 50 mM Tris–HCl buffer, pH 7.0, using a FLUOstar Optima plate reader (BMG LABTECH) with filters λex = 320 nm and λem = 420 nm. The reaction was initiated by the addition of 20 μM substrate. Unless stated otherwise, the incubation time was 5 min. The relative activity was estimated using a formula: (T-R)/(C-R)*100%, where T, C and R are test, control and reference samples respectively. The C sample refers to activity of the enzyme with no additive, and R refers to fluorescence background of the sample containing peptide only (no enzyme).

In order to analyze the effects of cations, the purified OpdA was subjected to treatment with 10 mM ethylene glycol tetraacetic acid (EGTA) for 10 min at room temperature followed by dialysis against PBS buffer for 2 h at room temperature. This treatment was repeated twice if required. The samples were incubated in 50 mM Tris–HCl buffer, (pH 7.0) supplemented with metal cations at required concentrations, at room temperature for 5 min.

To determine the effect of inhibitors on OpdA, the enzyme was first incubated with the inhibitor in 50 mM Tris–HCl buffer, pH 7.0 at room temperature for 5 min and the reaction initiated with 20 μM fluorogenic substrate. The inhibitors evaluated were 74 μM antipain, 1 mM EGTA, 0.1 mM chymostatin, 0.13 mM bestatin, 0.01 mM N-[N-(N-acetyl-L-leucyl)-L-leucyl]-L-norleucine (ALLN), 0.01 mM leupeptin, 1 mM PMSF, 1 mM 4-(2-aminoethyl) benzenesulfonyl fluoride (AEBSF), 28 μM E-64 and 0.01 mg/ml phosphoramidon according to manufacturer’s protocol (G-Biosciences). In order to study effects of pH on the enzymatic activity, the pH of the test buffer was adjusted with hydrochloric acid.

Statistical analysis of the results was carried out using a one way ANOVA. *P* value less than 0.05 was used to demonstrate statistically significant difference. Data were expressed as means ± SD of three readings from each of two independent experiments.

## Results

### The gene encoding OpdA of *Y. pseudotuberculosis* is required for virulence

In order to determine whether OpdA plays a role in pathogenesis in *Yersinia*, the gene encoding OpdA was disrupted in *Y. pseudotuberculosis* IP32953 (Additional file 1: Figure S1). Mice were then infected with mutant and wild-type *Y. pseudotuberculosis* and the CI was calculated. A CI value of 0.2 or less indicates that the locus is attenuating. The CI of the OpdA-defective mutant was 0.05, identifying OpdA as a potential virulence-associated protein. In contrast, no growth defect in vitro could be detected (Additional file 2: Figure S4).

### Purified OpdA protein reveals oligo-peptidase activity

**Fig. 1:**
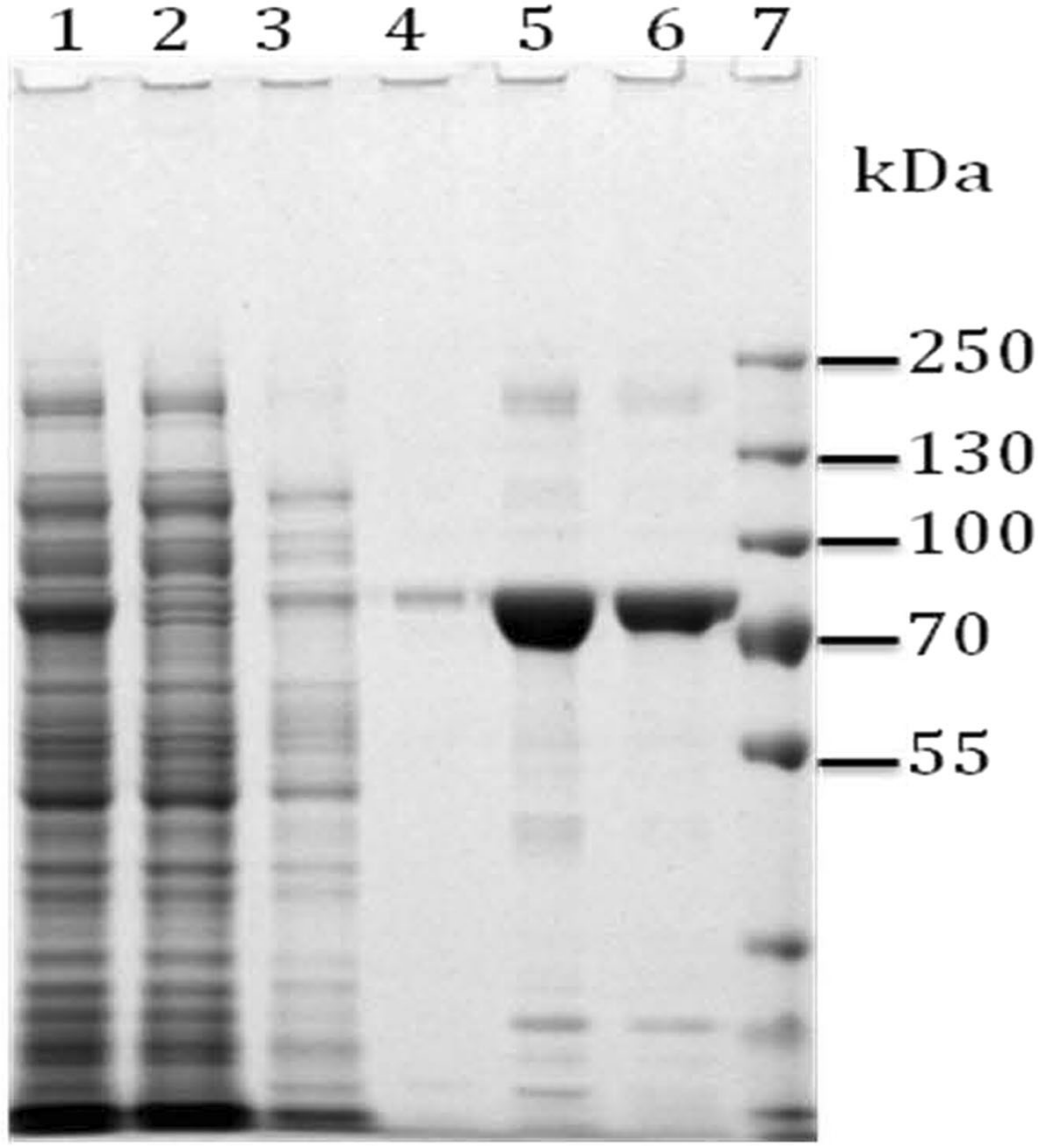
Purification of OpdA protein from *E. coli* XL1/pBAD33opdA-His strain after induction with L-arabinose. *Lane 1*, cell lysate; *lane 2*, flow-through; *lane 3*, wash 1; *lane 4*. wash 2, *lanes 5* and *6*, eluates; *lane 7* protein size markers (Fisher Scientific)

OpdA was expressed as an N-terminal 6xHis fusion protein and purified from *E.* c*oli*. Position of the recombinant protein on the gel (Fig. 1) fully corresponds to its molecular weight (77 kDa) estimated from the amino acid sequence. The protein was highly pure, stable and produced at a yield of 2 mg/l (Fig. 1).

No activity of the purified enzyme could be detected employing commercial protease assay (Protease Screening™ kit, Geno-Technology Inc) using casein as a substrate (data not shown). However, a very strong activity with Abz-NKPRRPQ-EDDnp substrate confirmed that the enzyme is an oligopeptidase. It was also found that, although EGTA inhibited the activity of the native enzyme (indicating that this was a metallopeptidase), a full inhibition of activity could not be achieved (Additional file 3: Figure S2). We reasoned that this could be due to extremely high affinity of the enzyme for metal cations, leaving residual amount of bound cations after a single treatment with EGTA. This hypothesis was confirmed by repeated treatment and dialysis, which resulted in a significant reduction of activity. Unless stated otherwise, the experiments described in this study were conducted with this enzyme ‘fully depleted’ of metal cations following dialysis treatment.

### Effect of pH on the activity of OpdA

**Fig. 2:**
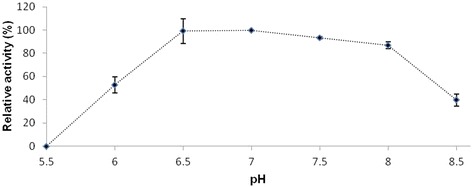
Effect of pH on OpdA activity. Abz-NKPRRPQ-EDDnp was used as the substrate. The activity at different pH values relative to that at pH 6.5 was determined. Mean values ± SD of three readings from each of two independent experiments are shown

Optimal pH range for OpdA activity with Abz-NKPRRPQ-EDDnp substrate was found to be between 6.0 and 8.0 (Fig. 2). The activities at pH 6.0 and pH 8.5 were 50 and 60% respectively when compared with the highest activity observed at pH 6.5.

### Effect of divalent cations on the hydrolytic activity of OpdA

**Fig. 3:**
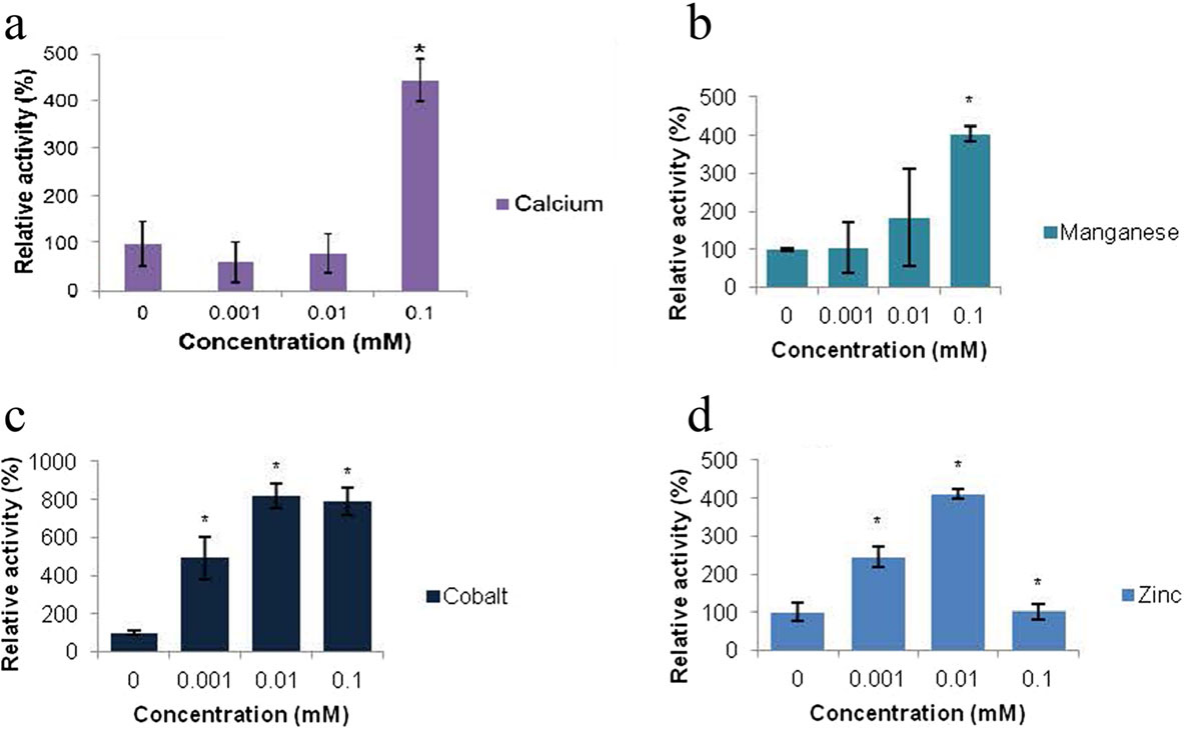
Effects of divalent cations on the hydrolysis of Abz-NKPRRPQ-EDDnp (20 μM) by OpdA. A *star* denotes a statistically significant difference compared to the test conducted in the absence of a cation. Mean values ± SD of three readings from each of two independent experiments are shown

The effect of different cations on the activity of OpdA and its ability to hydrolyze the substrate Abz-NKPRRPQ-EDDnp was evaluated (Fig. 3). Addition of Ca^2+^and Mn^2+^ did not produce any statistically significant change in activity at low concentration, but higher concentrations stimulated hydrolysis (Fig. 3a and b respectively). Addition of Co^2+^ stimulated hydrolysis at all concentrations (Fig. 3c) while Mg^2+^ and Cu^2+-^ did not show any stimulatory effect on activity of OpdA at any of the concentrations tested (data not shown). Zn^2+^, the proposed metal cofactor of OpdA, increased activity of the enzyme in the assay at low concentrations, but there appeared to be a limit to the concentration of Zn^2+^ the protein could tolerate, as the stimulation was abrogated at higher concentrations (Fig. 3e).

**Fig. 4:**
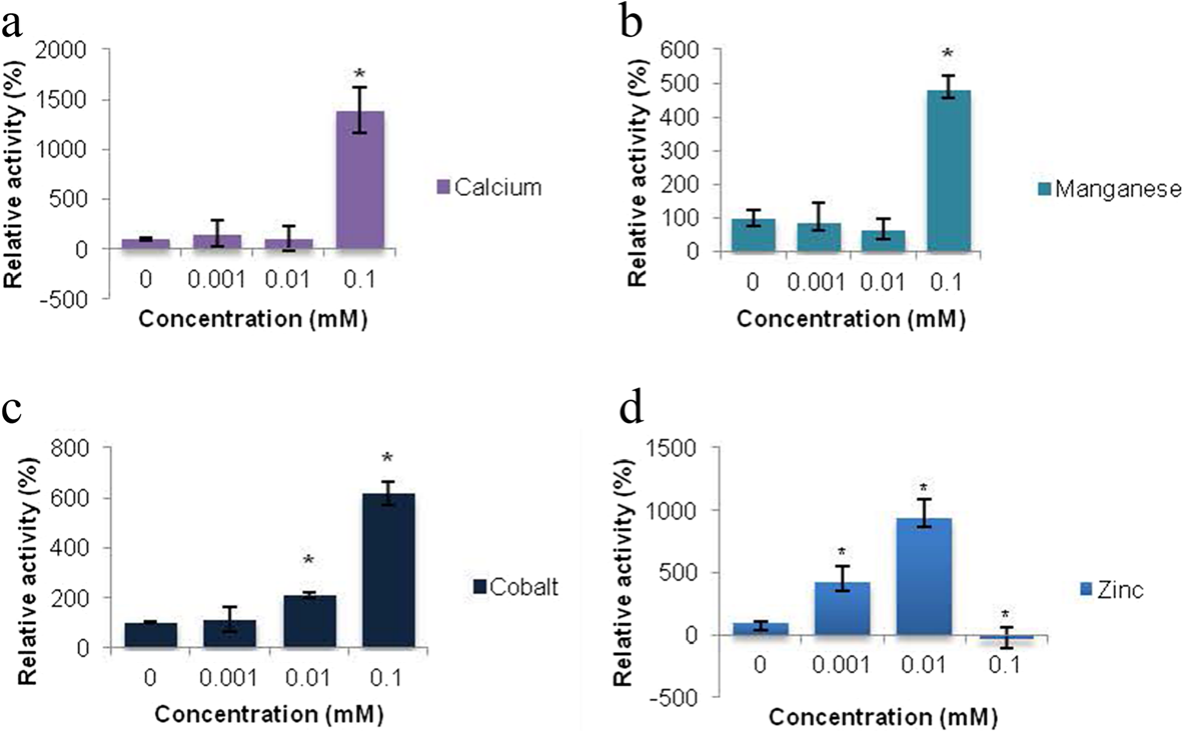
Effect of divalent cations on the hydrolysis of 20 μM Abz-AAL-EDDnp by double EGTA treated OpdA after 5 min. A *star* denotes a statistically significant difference compared to the test conducted in the absence of a cation. Mean values ± SD of three readings from each of two independent experiments are shown. Fluorescence (Y axis) is represented by arbitrary units

To determine whether the effects observed were substrate-specific, a second substrate, Abz-AAL-EDDnp was evaluated in the same way (Fig. 4).

Similar to a reaction with Abz-NKPRRPQ-EDDnp, the hydrolysis of this substrate required much higher concentrations of Ca^2+^and Mn^2+^ compared to Co^2+^ (Fig. 4a-c).

The enzyme was unaffected by the addition of Mg^2+^ and Cu^2+^ (data not shown). As with Abz-NKPRRPQ-EDDnp, the hydrolysis of Abz-NKPRRPQ-EDDnp was stimulated at low, but inhibited at high concentrations of Zn^2+^ (Fig. 4e).

**Fig. 5:**
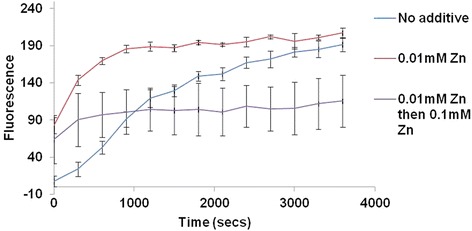
Effect of 0.1 mM Zn^2+^ on the activity of OpdA (0.4 μg) pre-treated with 0.01 mM of Zn^2+^Mean values ± SD of three readings from each of two independent experiments are shown. Fluorescence (Y axis) is represented by arbitrary units

**Fig. 6:**
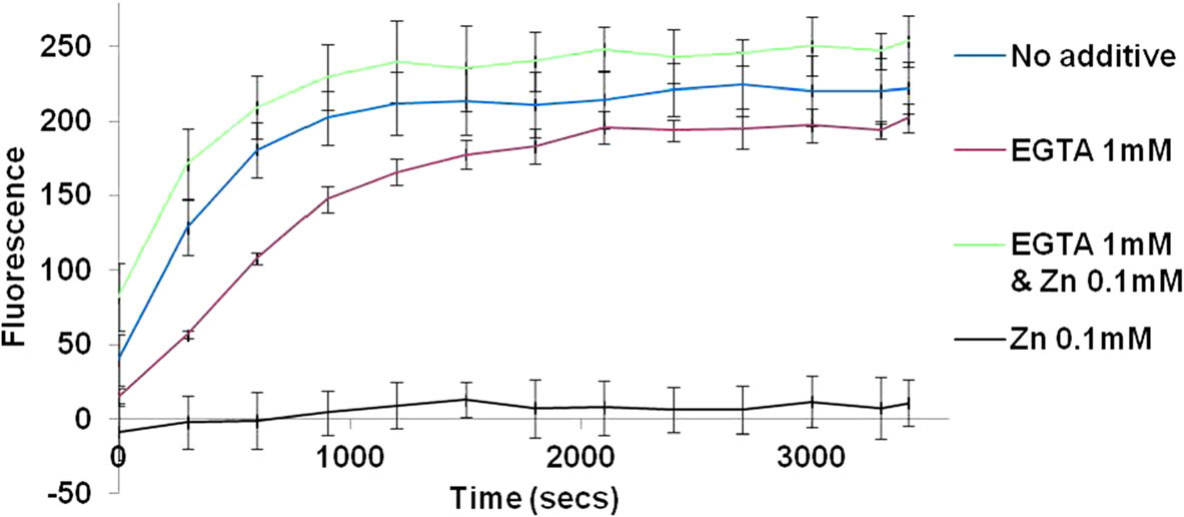
Effect of 0.1 mM Zn on native OpdA and the effect of 0.1 mM Zn on 1 mM EGTA treated OpdA (0.4 μg). Mean values ± SD of three readings from each of two independent experiments are shown. Fluorescence (Y axis) is represented by arbitrary units

To ascertain whether the inhibitory effect of zinc was due to steric changes in the active site following zinc binding, EGTA-treated OpdA was first incubated with 0.01 mM Zn^2+^ for 5 min, and then with the substrate Abz-NKPRRPQ-EDDnp for another 5 min at room temperature, to allow the reaction to proceed. This was followed by the addition of 0.1 mM Zn^2+^ in an attempt to inhibit the enzyme. The reaction was transferred to 37 °C and the hydrolysis of the peptide was analyzed for an hour. The results demonstrated the inhibition of hydrolysis after the addition of 0.1 mM Zn^2+^ (Fig. 5). To determine whether the inhibition was reversible, non-EGTA treated OpdA was first incubated with 0.1 mM Zn^2+^ for 5 min, then with 1 mM EGTA. The reaction was initiated with 20 μM Abz-NKPRRPQ-EDDnp substrate and incubated at 37 °C. EGTA was able to reverse the inhibitory effect of excess Zn^2+^ (Fig. 6).

### Effect of inhibitors on the hydrolytic activity of OpdA

The effect of inhibitors on OpdA was evaluated. Protein preparations were used that had been purified from *E. coli* as above, but not treated with EGTA. Enzyme activity was evaluated after 5 min pre-incubation with the inhibitor, using Abz-NKPRRPQ-EDDnp as the substrate (Table 1). The activity of OpdA was not affected by phosphoramidon, E-64, bestatin, PMSF, AEBSF and leupeptin, but was reduced in the presence of antipain, chymostatin, ALLN and EGTA. The activity of OpdA was not affected by phosphoramidon, bestatin, PMSF, AEBSF and leupeptin, but was decreased in the presence of antipain, chymostatin, ALLN and EGTA. A similar effect of inhibitors on enzyme activity was observed when using the second substrate, Abz-AAL-EDDnp (Table 2).

**Table 1:** Effect of inhibitors on the hydrolysis of Abz-NKPRRPQ-EDDnp by OpdA

Inhibitor	Relative activity (%)	*P* Value
None	100	
Phosphoramidon	92 ± 9	0.5759
E-64	101 ± 3	1.0000
Bestatin	95 ± 22	0.7791
PMSF	92 ± 14	0.4685
AEBSF	99 ± 10	0.9252
Leupeptin	87 ± 8	0.4590
Antipain	31 ± 2	0.0002*
ALLN	45 ± 9	0.0127*
Chymostatin	39 ± 11	0.0028*
EGTA	73 ± 9	0.0228*

A star (*) denotes statistically valid difference (*P* < 0.05) compared to control (no inhibitors)

**Table 2:** Effect of inhibitors on the hydrolysis of Abz-AAL-EDDnp by OpdA

Inhibitor	Relative activity (%)	*P* Value
None	100	-
Phosphoramidon	150 ± 66	0.3515
E-64	212 ± 129	0.2361
Bestatin	103 ± 31	1.0000
PMSF	148 ± 61	0.3053
AEBSF	82 ± 12	0.3262
Leupeptin	73 ± 7	0.1690
Antipain	28 ± 5	0.0008*
ALLN	1 ± 10	0.0004*
Chymostatin	4 ± 34	0.0077*
EGTA	3 ± 16	0.0175*

A star (*) denotes statistically valid difference (*P* < 0.05) compared to control (no inhibitors)

## Discussion

Deletion of *opdA* gene in *Y. pseudotuberculosis* IP32953 resulted in attenuation of this strain in a mouse model of infection, suggesting a possible role of the OpdA protein in infection. In this study we confirmed that this protein as an oligopeptidase and tested effects of pH, various cations and peptidase inhibitors on its activity using two substrates. OpdA from *S.* Typhimurium and *E. coli* were shown to be able to use a broad range of oligopeptide substrates, but no preference was reported [1, 7, 8, 10, 17]. Although OpdA belongs to the M3A subfamily of Zn-dependent metallo-proteases [7, 8, 10], in the current study the enzyme was able to fully hydrolyze the substrates in the absence of exogenously added Zn^2+^ possibly due to trace cations present in the OpdA active site.

Previous reports demonstrated inhibition of *S.* Typhimurium OpdA by Zn^2+^ at 0.1 mM [3, 17]. Our data support this result. We investigated this phenomenon in more detail by using OpdA depleted of naturally bound divalent cations by repeated treatment with EGTA followed by dialysis (in order to remove EGTA and soluble EGTA-complexes). In these samples we found stimulation of OpdA activity by low Zn^2+^ concentrations (10 μM), whilst there was complete inhibition at 100 mM of this cation. As observed previously with OpdA from *S. typhimurium* [6, 17], cobalt, calcium and manganese cations stimulated the activity of OpdA. The ability of OpdA to utilize different metal ions for hydrolysis is possibly due to the co-ordination geometries of these metals and the flexibility of the active center [18].

A previous study suggested a possibility of OpdA from *E. coli* having two active sites [3]. In particular, that study showed stimulation of OpdA activity by Co^2+^ when hydrolysing Z-AALpNA, but slight inhibition when hydrolysing Boc-Val-Pro-Arg-NH-Mec. In addition, inhibition assays from the study showed that the hydrolysis of Z-AALpNA was not affected by antipain or 4-guanidino benzoic acid 4- tert-butylphenyl ester, whereas the hydrolysis of Boc-Val-Pro-Arg-NH-Mec was completely inhibited by these two inhibitors.

Following these data reported by Jiang et al. [3], in the current study we decided to examine the ‘trypsin-like’ activity of OpdA by using Abz-NKPRRPQ-EDDnp as it has two arginine residues, which trypsin could cleave [19]. However, we found that the hydrolysis of Abz-NKPRRPQ-EDDnp by OpdA was not completely inhibited by trypsin specific inhibitor antipain and the enzyme was able to fully hydrolyze the substrate within an hour. In contrast to data by Jiang et al. [3] describing *E. coli* OpdA-like protein (named PrlC, or rPrlC for its ‘recombinant’ form), in our experiments an addition of Co^2+^ to partially depleted OpdA of *Y. pseudotuberculosis* stimulated the hydrolysis of both Abz-NKPRRPQ-EDDnp and Abz-AAL-EDDnp. The discrepancies in the results may be due to different properties of OpdA proteins extracted from different sources. Neither our data with two different substrates, nor more recent studies provide any evidence for the presence of two active centers in OpdA and similar enzymes [1, 7, 8]. Putative amino acid residues involved in catalytic activity of *Y. pseudotuberculosis* OpdA by similarity to *E. coli* OpdA are showing in Additional file 4: Figure S3.

Previous studies showed the inhibitory effect of EDTA on the hydrolysis of AcAla_4_ by OpdA [6, 17]. In contrast, our initial experiments with the hydrolysis of Abz-NKPRRPQ-EDDnp using native (untreated) OpdA showed only partial inhibition of activity in the presence of 1 mM EDTA (data not shown). Increasing concentration of the latter to 5 mM did not increase the inhibitory effect. No statistically valid difference in the latter was observed when EDTA was replaced with EGTA. This could be due to much higher affinity of divalent cations to OpdA than to EDTA or EGTA. We managed to alleviate the problem with partial inhibition by using sequential double treatment of the enzyme with EGTA followed by dialysis.

Despite confirming that OpdA is a metallopeptidase, unexpectedly we found inhibitory effects of antipain and chymostatin. This could be a result of disturbances to the flexible loop i.e. ^600^SHIFAGGYAAGYYSY^614^. The flexible loop contains two serine residues that could be important for enzyme activity and may be the site of action by antipain and chymostatin. It is also noteworthy that the loop also contains the important substrate recognition residue Tyr607 [7].

OpdA and Dcp are thought to be the major peptidases involved in the hydrolysis of peptides in the cytoplasm since bacterial lysates lacking both of these enzymes have no catalytical activity against AcAla_3_ and AcAla_4_ [6]. Incubation of both Abz-GFSPFR-EDDnp and Abz-GFSPFRQ-EDDnp by OpdA with *E. coli* bacterial lysate produced the Abz-GF product suggesting that OpdA is the major oligopeptidase in *E. coli* [8]. Although another study also demonstrated intracellular location of OpdA [9], one can’t exclude a possibility of extracellular release of this enzyme during infection.

A role of OpdA protein in pathogenesis remains to be elucidated. One attractive hypothesis is a possibility of this enzyme destroying short positively charged antimicrobial peptides involved in innate host immunity. Remarkably, the substrate used in this study is derived from bradykinin peptide known for its antimicrobial activity [20]. One of the mechanisms of bacterial resistance to antimicrobial peptides is degradation of these compounds by oligopeptidases [21].

Overall, the current study has demonstrated the effect of various metal ions and inhibitors on the hydrolysis of two fluorogenic substrates by OpdA. The study showed concentration dependent inhibitory effect of zinc on OpdA activity. Further research could be aimed at determining the residues responsible for the zinc inhibition via site directed mutagenesis. Furthermore, it will be interesting to determine if replacement of the wild type copy of the *opdA* gene with that encoding a modified protein (e.g. not inhibited by elevated Zn^2+^ concentrations) would have any effect on the virulence of *Y. pseudotuberculosis*.

## Conclusions

In this study we report the identification and enzymatic properties of *Yersinia pseudotuberculosis* oligopeptidase (OpdA) required for bacterial pathogenicity. The enzyme was found to be specific to short oligopeptides, with its peptidase activity dependent on the presence of particular divalent cations. The activity of this enzyme towards positively charged peptides mimicking those produced by host innate immune system may explain the reason for attenuation of the *opdA* mutant of this bacterium.

The data generated by this study are novel and important as they:

- present results of extensive studies on the effects of different cations of the activity of this enzyme.
- explain unexpected limited effects of chelators on the native enzyme predicted to be a metallopeptidase.
- describe a novel approach used for ‘depletion’ of the native enzyme of divalent cation(s) by repeated treatment with a chelator and dialysis.
- show unusual and unexpected inhibitory effect of higher concentrations of Zn^2+^ on the activity.
- demonstrate that the latter could be observed even after initial activation of the enzyme at lower Zn^2+^ concentrations.
- show that the inhibitory effect is reversible due to unexpected restoration of activity after addition of EGTA.

In addition, this is the first study suggesting a role of OpdA enzyme in pathogenicity. The results reported provide valuable contribution to the investigation of bacterial metallopeptidases in general, and that of oligopeptidases in particular, and are important for better understanding of the role of OpdA-like enzymes in pathogenesis of bacterial infections. Furthermore, characterisation of this protein may serve as a basis for the development of novel antibacterials based on specific inhibition of its peptidase activity.

## Additional files

Additional file 1: Figure S1. PCR analysis of *Y. psedutuberculosis* YPIII wild type (lane 2), and three clonal isolates of the Δ*opdA* mutant (lanes 3–5) using gene specific primers Yptb3816_for (ATGACAAACCCGCTGTTGACT) and Yptb3816_rev (TTAGCCCTTAATACCGTAATGAC). The expected product size after deletion is 1.2 kb, corresponding to a deletion of almost the entire gene sequence (1.9 out of 2.1 kb) plus 1 kb corresponding to the inserted *kan* ^*r*^ resistance gene. Lane 1, DNA size ladder 1 kb plus (Life Technologies). (JPG 69 kb)
Additional file 2: Figure S4. Comparison of growth rates of the wild strain of *Y. psedotuberculosis* IP32953 and its *opdA* mutant (opdA). Y axis, optical density (OD_595_); X axis, time (hours); *n* = 18 (3 biological and 6 technical replicates. (JPG 22 kb)
Additional file 3: Figure S2. A representative experiment demonstrating the lack of complete inhibition of the activity of native OpdA enzyme (0.4 μg, green triangles) in the presence of EGTA (1 mM, blue crosses). (JPG 92 kb)
Additional file 4: Figure S3. Multiple alignment of amino acid sequences of OpdA proteins found in different bacteria: *Y. pseudotuberculosis* (accession number CAH23054.1), *E. coli* (synonym name PrlC, accession number P27298.3), *Salmonella enterica* serovar Typhimurium (accession number P27237.1) and *Haemophilus influenzae* (accession number P44573.1). Amino acid residues corresponding to Zn^2+^ binding motif (by similarity to *E. coli* OpdA) are highlighted in red. A Tyr residue corresponding to Tyr607 of *E. coli* OpdA shown to be essential for enzyme activity and specificity is also highlighted (in green). (JPG 525 kb)

## Abbreviations

Abz: O-aminobenzoyl, fluorophore
EDDnp: Ethylenediamine 2,4-dinitrophenyl, quencher
OpdA: Oligopeptidase A
