# Supplementary figures and images for: YPTB3816 of Yersinia pseudotuberculosis strain IP32953 is a virulence-related metallo-oligopeptidase
Source: BMC Microbiol. 2016 Nov 25;16:282. doi: 10.1186/s12866-016-0900-7 (PMC5124237; doi:10.1186/s12866-016-0900-7)

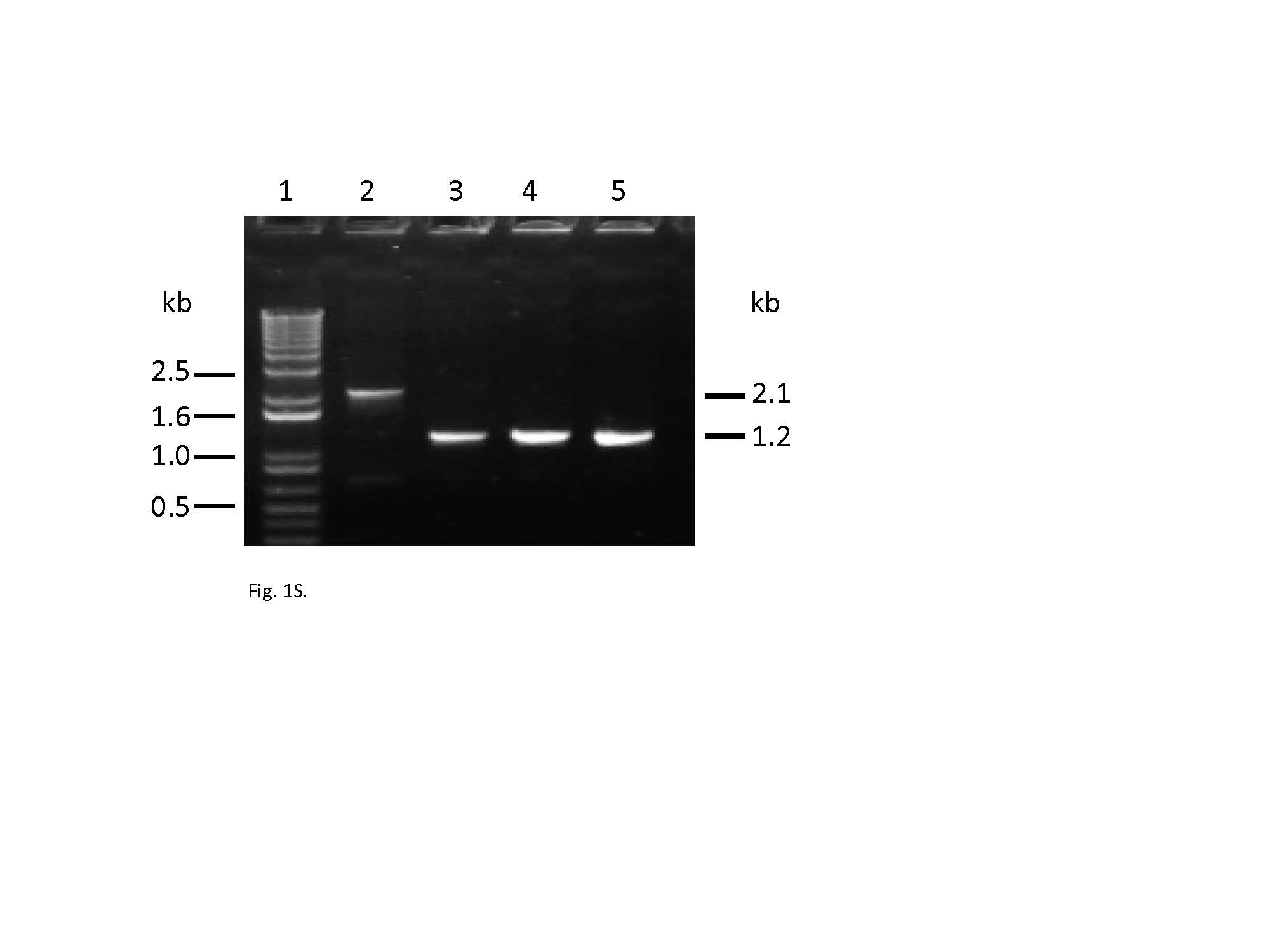

Supplement: Additional file 1: Figure S1. — PCR analysis of Y. psedutuberculosis YPIII wild type (lane 2), and three clonal isolates of the ΔopdA mutant (lanes 3–5) using gene specific primers Yptb3816_for (ATGACAAACCCGCTGTTGACT) and Yptb3816_rev (TTAGCCCTTAATACCGTAATGAC). The expected product size after deletion is 1.2 kb, corresponding to a deletion of almost the entire gene sequence (1.9 out of 2.1 kb) plus 1 kb corresponding to the inserted kan r resistance gene. Lane 1, DNA size ladder 1 kb plus (Life Technologies). (JPG 69 kb) [file 12866_2016_900_MOESM1_ESM.jpg]

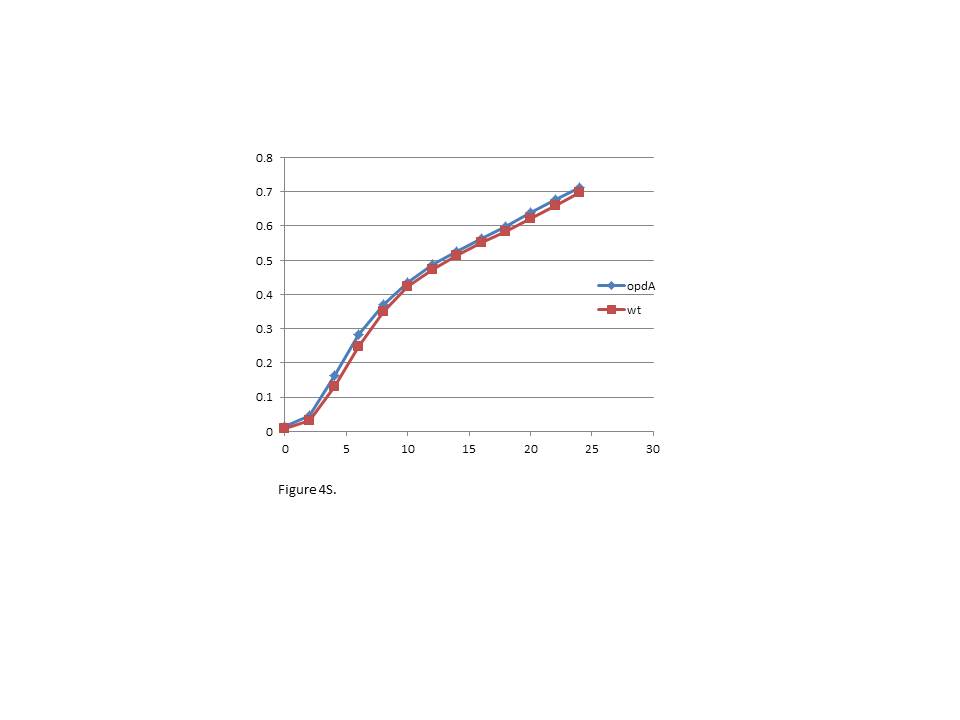

Supplement: Additional file 2: Figure S4. — Comparison of growth rates of the wild strain of Y. psedotuberculosis IP32953 and its opdA mutant (opdA). Y axis, optical density (OD595); X axis, time (hours); n = 18 (3 biological and 6 technical replicates. (JPG 22 kb) [file 12866_2016_900_MOESM2_ESM.jpg]

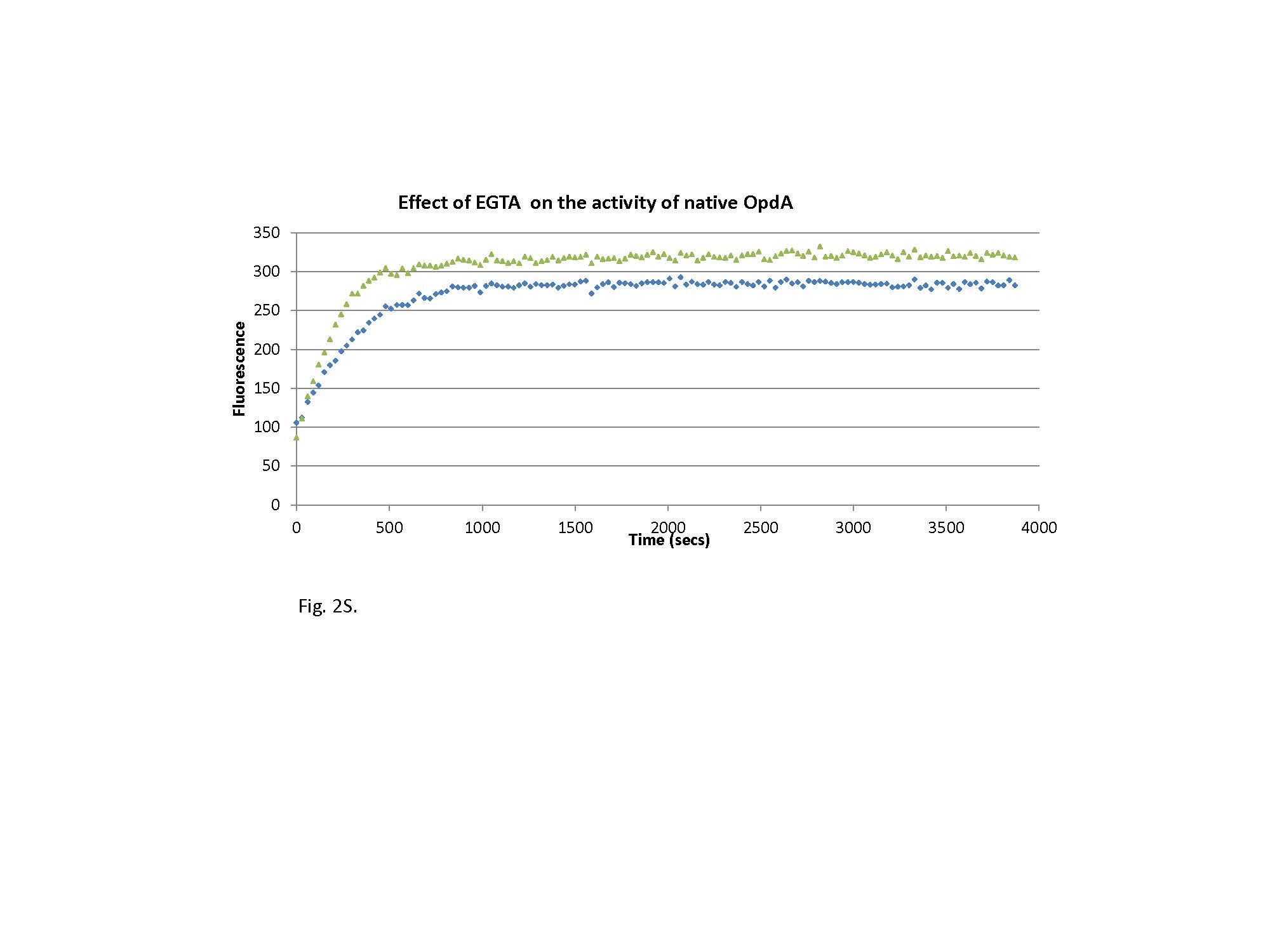

Supplement: Additional file 3: Figure S2. — A representative experiment demonstrating the lack of complete inhibition of the activity of native OpdA enzyme (0.4 μg, green triangles) in the presence of EGTA (1 mM, blue crosses). (JPG 92 kb) [file 12866_2016_900_MOESM3_ESM.jpg]

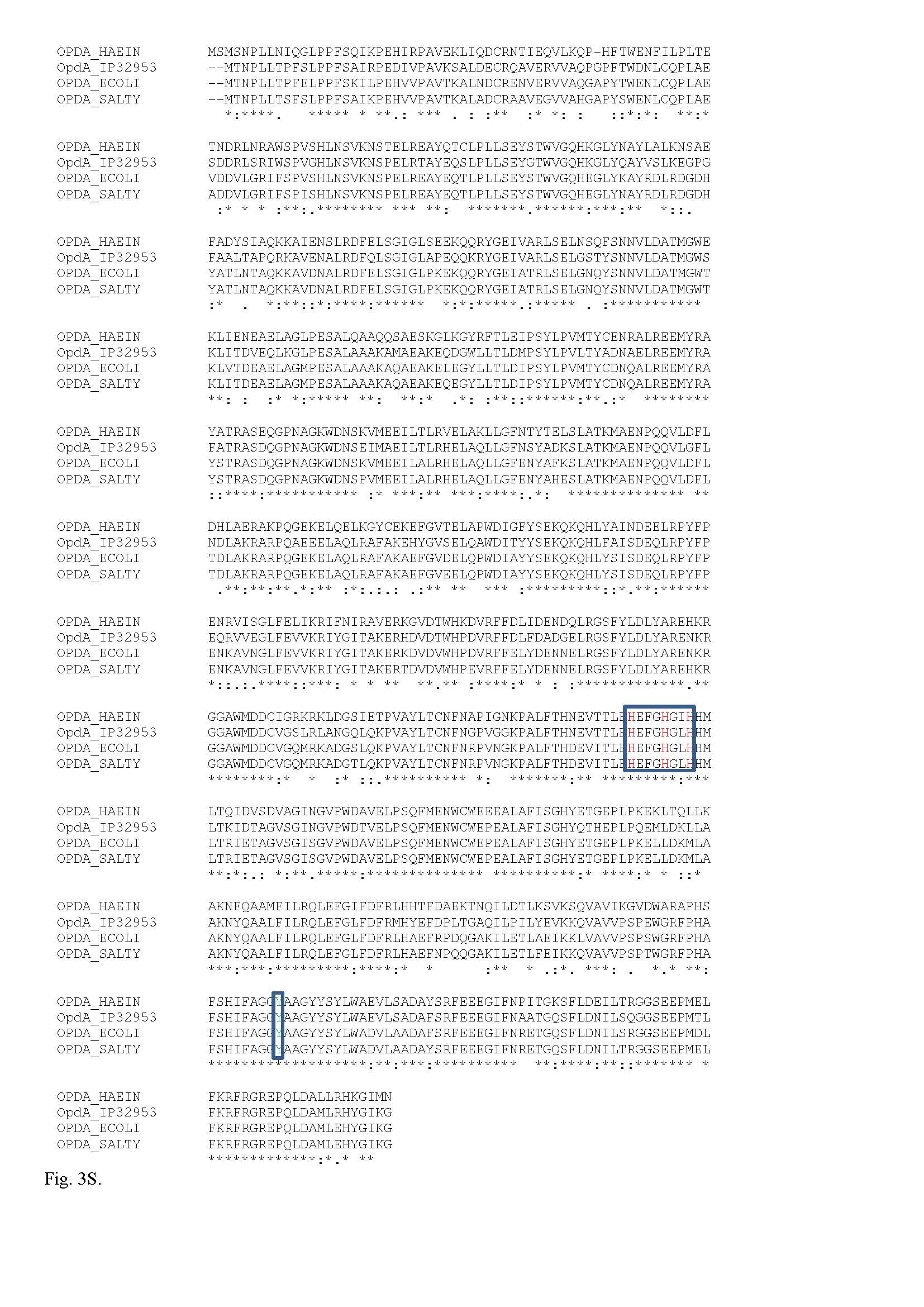

Supplement: Additional file 4: Figure S3. — Multiple alignment of amino acid sequences of OpdA proteins found in different bacteria: Y. pseudotuberculosis (accession number CAH23054.1), E. coli (synonym name PrlC, accession number P27298.3), Salmonella enterica serovar Typhimurium (accession number P27237.1) and Haemophilus influenzae (accession number P44573.1). Amino acid residues corresponding to Zn2+ binding motif (by similarity to E. coli OpdA) are highlighted in red. A Tyr residue corresponding to Tyr607 of E. coli OpdA shown to be essential for enzyme activity and specificity is also highlighted (in green). (JPG 525 kb) [file 12866_2016_900_MOESM4_ESM.jpg]
